# *Helicobacter pylori* infection is associated with diabetes among Chinese adults

**DOI:** 10.1111/jdi.13102

**Published:** 2019-07-04

**Authors:** Zhengce Wan, Lulu Song, Liu Hu, Mei Hu, Xiaomei Lei, Yuancheng Huang, Yongman Lv

**Affiliations:** ^1^ Physical Examination Center Tongji Hospital Tongji Medical College Huazhong University of Science and Technology Wuhan China; ^2^ Department of Maternal and Child Health School of Public Health Tongji Medical College Huazhong University of Science and Technology Wuhan China

**Keywords:** Diabetes, Glycemic metabolism, *Helicobacter pylori* infection

## Abstract

**Aims/Introduction:**

Several epidemiological studies investigated the effect of *Helicobacter pylori* infection on diabetes, but the conclusions remained inconsistent. We aimed to explore the relationship between *H. pylori* infection and diabetes, as well as glycemic metabolism profiles.

**Materials and Methods:**

A cross‐sectional study including 58,482 Chinese adults was carried out between January 2016 and December 2017. *H. pylori* infection was diagnosed by the ^13^C‐urea breath test. Multivariate regression analyses were carried out to evaluate the association of *H. pylori* infection with diabetes.

**Results:**

Of the 58,482 participants, 3,449 (5.9%) had diabetes. The *H. pylori*‐positive participants had a higher rate of diabetes (7.3% vs 5.2%, *P* < 0.001), and higher levels of fasting plasma glucose (5.36 ± 1.12 mmol/L vs 5.28 ± 0.95 mmol/L, *P* < 0.001) and glycated hemoglobin A1c (5.63 ± 0.68% vs 5.57 ± 0.60%, *P* < 0.001) than the *H. pylori* negative group. Multivariate regression analyses showed that *H. pylori* infection was positively related to diabetes (odds ratio 1.25, 95% confidence interval 1.15–1.35). Among the *H. pylori*‐positive participants, the elevated levels of fasting plasma glucose and glycated hemoglobin A1c were 0.033 mmol/L (95% confidence interval 0.016–0.049 mmol/L) and 0.024% (95% confidence interval 0.008–0.041%), respectively. Additionally, *H. pylori* infection was significantly related to diabetes in participants aged ≥44 years, but not in participants aged <44 years.

**Conclusions:**

The present study showed that *H. pylori* infection is associated with diabetes among Chinese adults. More attention should be paid to adults with *H. pylori* infection for effective prevention of diabetes.

## Graphical abstract

We found that *Helicobacter pylori* infection was independently associated with a higher prevalence of diabetes among Chinese adults. More attention should be paid to adults with *H. pylori* infection for effective prevention of diabetes.

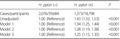

## Introduction

The number of diabetes patients worldwide has increased dramatically during the past few decades, especially in developing countries^1^. In 2017, China had the world's largest diabetic population, with a total of 114.4 million adult patients^2^. A nationally representatively survey in mainland China showed that the estimated prevalence of diabetes was 10.9% among Chinese adults^3^. Diabetes seriously affects the patients’ quality of life, and significantly increases the risk of all‐cause mortality among adults in both rural and urban areas of China^4^, ^5^. Risk factors for diabetes, such as genetics, adiposity and smoking, have been well established in past decades. However, whether infectious pathogens are involved in the pathogenesis of diabetes remains inconclusive. In the present study, we focused on the effect of *Helicobacter pylori* infection on diabetes.

*H. pylori*, a kind of Gram‐negative bacteria that colonizes the stomach of humans, infects 44.3% of the world's population^6^. *H. pylori* generally causes gastrointestinal diseases, such as chronic gastritis, peptic ulcer disease and gastric malignancy. Epidemiological evidence showed that *H. pylori* infection also increased the risk of chronic metabolic diseases, such as obesity, dyslipidemia and hypertension^7^, ^8^, ^9^. A meta‐analysis including 41 studies documented that diabetes was significantly associated with an elevated rate of *H. pylori* infection^10^, indicating that diabetes could promote susceptibility to *H. pylori* infection. However, does *H. pylori* infection inversely have an impact on diabetes development? Several studies explored the effect of *H. pylori* infection on diabetes, but the conclusions remained inconsistent^11^, ^12^, ^13^, ^14^, ^15^, ^16^. A prospective study of 782 Latinos aged >60 years reported a positive association of *H. pylori* infection with diabetes^11^, whereas a cross‐sectional study of 1,000 multiethnic Americans aged 45–84 years showed no significant association between them^12^; both of the two studies had a relatively small sample size and used a serological test to diagnose *H. pylori* infection. Serological antibody for *H. pylori* might last for several years after successful eradication of *H. pylori* 17, which means that the serological test is not able to adequately represent the current *H. pylori* infection status.

We carried out the present large cross‐sectional study to examine the relationship between *H. pylori* infection, as diagnosed by the ^13^C‐urea breath test, and diabetes as well as glycemic metabolism profiles, including fasting plasma glucose (FPG) and glycated hemoglobin A1c (HbA1c), in a Chinese population.

## Methods

### Study population

We used data from a cross‐sectional study, which was carried out in the physical examination center of Tongji hospital in Wuhan city, Hubei province, China. Participants aged ≥18 years who had a health checkup in the study hospital were recruited between January 2016 and December 2017. Most of the participants were urban residents employed by government or enterprises. The health checkup data included demographic characteristics, serum biochemical index, anthropometric parameters, the ^13^C‐urea breath test and information on medical histories. Finally, a total of 58,482 participants who underwent both the ^13^C‐urea breath test and the monitoring of glycemic metabolism profiles were included in the current study.

The ethics committee of Tongji Hospital, Tongji Medical College, Huazhong University of Science and Technology approved the present study (institutional review board approval number: TJ‐C20160115). The study conformed to the ethical guidelines of the Declaration of Helsinki. Written informed consent was collected from each participant.

### Assessment of diabetes

Fasting plasma glucose was measured by the hexokinase method using the Cobas 8000 modular analyzer (Roche Diagnostics, Basel, Switzerland). HbA1c was measured by the ion‐exchange high‐performance liquid chromatography method using an automatic glycohemoglobin analyzer (ADAMS A1c, HA‐8180; ARKRAY, Kyoto, Japan). In the current study, diabetes was assessed according to the American Diabetes Association criteria^18^ as meeting any of the following criteria: (i) FPG ≥7.0 mmol/L; (ii) HbA1c ≥6.5%; and (iii) as the participants who self‐reported physician diagnosis of diabetes with use of antidiabetic medication (hypoglycemic agent or insulin) might have normal levels of FPG and HbA1c, they were also considered as diabetics in the present study.

### Diagnosis of *H. pylori* infection

The ^13^C‐urea breath test was applied to diagnose *H. pylori* infection. Participants who had the ^13^C‐urea breath test were overnight fasting for at least 8 h. Participants were required to take 75 mg of ^13^C‐urea (Urea‐^13^C Capsule Breath Test Kit; HEADWAY, Shenzhen, China) after providing the initial baseline breath sample. We collected the second breath sample after half an hour. The samples were detected using a ^13^C‐urea breath test analyzer (HCBT‐01; HEADWAY). *H. pylori* infection was defined as positive if the difference between the two samples exceeded 4.0 parts per 1,000 of ^13^CO_2_. Otherwise, it was considered negative.

### Assessment of covariates

Demographic characteristics (age, sex) and medical histories were collected by a questionnaire. Standard laboratory methods were applied to obtain data of triglyceride, total cholesterol, low‐density lipoprotein, high‐density lipoprotein, uric acid and creatinine. Hypertension was defined as follows: systolic blood pressure ≥140 mmHg, or diastolic blood pressure ≥90 mmHg, or use of antihypertensive medication, or self‐reported physician diagnosis of hypertension. Body mass index (BMI) was calculated as weight in kilograms divided by the square of height in meters.

### Statistics analysis

In the current study, SPSS version 17.0 (SPSS Inc., Chicago, IL, USA) was applied to carry out all statistical analyses. Continuous and categorical variables were presented as means ± standard deviation and percentages, respectively. Differences in the data were evaluated by Student's *t*‐tests and χ^2^‐tests accordingly. Regression coefficients (β) and their 95% confidence intervals (CI) from multivariate linear regression models were used to evaluate the association of *H. pylori* infection with FPG and HbA1c. Of the 58,482 participants, 54,404 (93%) were involved as no antidiabetic medication use. We did not include participants who took antidiabetic medication when calculating the average glucose metabolism levels and carrying out the multivariate linear regression analyses, as they might have normal levels of FPG and HbA1c. Logistic regression models were used to assess the association between *H. pylori* infection and diabetes. The results were presented as odds ratios (ORs) and 95% CIs. Model 1 adjusted for age and sex. In model 2, BMI was added. Model 3 further included blood lipids (triglyceride, total cholesterol, low‐density lipoprotein, high‐density lipoprotein), hypertension, uric acid and creatinine. We also carried out stratified analyses according to several major confounders. Age was stratified by the median value (44 years). BMI was stratified by the cut‐off value of overweight for Asian populations (24 kg/m^2^). We then carried out the Wald test to explore the interaction across the subgroup. The *P* for interaction was tested for multiplicative interactions. A two‐sided *P‐*value of <0.05 was considered statistically significant.

## Results

Of the 58,482 participants (21,867 women and 36,615 men), the mean age was 43.81 ± 11.52 years. The rate of diabetes was 5.9%. The percentage of *H. pylori* infection in diabetes patients was significantly higher than that in non‐diabetic participants (39.8% vs 31.7%, *P* < 0.05). There were 18,798 (32.1%) participants infected with *H. pylori*. The participants’ characteristics according to the status of *H. pylori* infection are presented in Table 1. The *H. pylori*‐positive participants had a higher proportion of diabetes and hypertension (all *P* < 0.001). The levels of age, BMI, blood pressure, triglyceride, total cholesterol, low‐density lipoprotein, FPG and HbA1c were higher in participants infected with *H. pylori* (all *P* < 0.001). In addition, the level of high‐density lipoprotein was lower in participants with *H. pylori* infection (*P* < 0.001).

**Table 1:** Characteristics of participants according to the status of *Helicobacter pylori* infection

Variable	*H. pylori* (+)	*H. pylori* (−)	*t/*χ^2^	*P*
Age (years)	44.85 ± 11.26	43.32 ± 11.62	−15.20	<0.001
Female, *n* (%)	6,999 (37.2)	14,868 (37.5)	0.30	0.585
BMI (kg/m^2^)	24.32 ± 3.32	23.93 ± 3.35	−12.88	<0.001
SBP (mmHg)	125.17 ± 18.61	123.64 ± 17.67	−9.26	<0.001
DBP (mmHg)	77.29 ± 12.48	76.35 ± 12.11	−8.46	<0.001
Triglyceride (mmol/L)	1.60 ± 1.40	1.54 ± 1.33	−4.56	<0.001
TC (mmol/L)	4.65 ± 0.89	4.58 ± 0.86	−8.06	<0.001
HDL (mmol/L)	1.24 ± 0.29	1.26 ± 0.30	9.32	<0.001
LDL (mmol/L)	2.82 ± 0.76	2.76 ± 0.74	−8.96	<0.001
Creatinine (umol/L)	74.92 ± 17.39	74.67 ± 20.36	−1.45	0.147
Uric acid (umol/L)	348.12 ± 95.91	346.62 ± 95.20	−1.77	0.076
FPG (mmol/L)	5.36 ± 1.12	5.28 ± 0.95	−8.43	<0.001
HbA1c (%)	5.63 ± 0.68	5.57 ± 0.60	−6.67	<0.001
Diabetes, *n* (%)	1,373 (7.3)	2,076 (5.2)	98.74	<0.001
Hypertension, *n* (%)	4,907 (27.1)	9,097 (23.8)	71.44	<0.001

Data are the mean + standard deviation or percentages. BMI, body mass index; DBP, diastolic blood pressure; FPG, fasting plasma glucose; *H. pylori*,* Helicobacter pylori*; HbA1c, glycated hemoglobin A1c; HDL, high‐density lipoprotein; LDL, low‐density lipoprotein; SBP, systolic blood pressure; TC, total cholesterol.

Table 2 shows the association of *H. pylori* infection with FPG and HbA1c using multiple linear regression models. In model 1, which adjusted for age and sex, *H. pylori* infection was related to higher levels of FPG and HbA1c (all *P* < 0.001). Additional adjustment for BMI in model 2 did not alter the positive association (all *P* < 0.001). In model 3, which further included hypertension, blood lipids, uric acid and creatinine, the positive association of *H. pylori* infection with FPG and HbA1c remained significant; among the *H. pylori*‐positive participants, the elevated levels of FPG and HbA1c were 0.033 mmol/L (95% CI 0.016–0.049) and 0.024% (95% CI 0.008–0.041), respectively.

**Table 2:** Association of *Helicobacter pylori* infection with levels of fasting plasma glucose and hemoglobin A1c

	FPG		HbA1c (%)	
	β (95% CI)	*P*	β (95% CI)	*P*
Unadjusted	0.081 (0.063–0.098)	<0.001	0.062 (0.045–0.080)	<0.001
Model 1	0.055 (0.038–0.073)	<0.001	0.046 (0.029–0.063)	<0.001
Model 2	0.038 (0.021–0.055)	<0.001	0.032 (0.016–0.049)	<0.001
Model 3	0.033 (0.016–0.049)	<0.001	0.024 (0.008–0.041)	0.003

Model 1: adjusted for age and sex. Model 2: adjusted for age, sex and body mass index. Model 3: adjusted for age, sex, body mass index, triglyceride, total cholesterol, low‐density lipoprotein, high‐density lipoprotein, uric acid, creatinine and hypertension. CI, confidence interval; FPG, fasting plasma glucose; *H. pylori*,* Helicobacter pylori*; HbA1c, hemoglobin A1c.

The association of *H. pylori* infection with diabetes is shown in Table 3. *H. pylori* infection was associated with an increased risk of diabetes after controlling for age and sex (model 1), with an OR of 1.34 (95% CI 1.25–1.44). After additional adjustment for BMI (model 2), the positive association between them was similarly observed (OR 1.28, 95% CI 1.19–1.38). The fully adjusted analysis (model 3) suggested that the positive association between *H. pylori* infection and the risk of diabetes did not materially change (OR 1.25, 95% CI 1.15–1.35).

**Table 3:** Effects of *Helicobacter pylori* infection on diabetes

	*H. pylori* (−)	*H. pylori* (+)	*P*
Cases/participants	2,076/39,684	1,373/18,798	
Unadjusted	1.00 (Reference)	1.43 (1.33, 1.53)	<0.001
Model 1	1.00 (Reference)	1.34 (1.25, 1.44)	<0.001
Model 2	1.00 (Reference)	1.28 (1.19, 1.38)	<0.001
Model 3	1.00 (Reference)	1.25 (1.15, 1.35)	<0.001

Data presented as odds ratios and 95% confidence intervals. Model 1: adjusted for age and sex. Model 2: adjusted for age, sex and body mass index. Model 3: adjusted for age, sex, body mass index, triglyceride, total cholesterol, low‐density lipoprotein, high‐density lipoprotein, uric acid, creatinine and hypertension. *H. pylori*,* Helicobacter pylori*.

Age, sex and BMI were the major risk factors. Subgroup analyses were carried out according to these three risk factors (Table 4). *H. pylori* infection was significantly related to diabetes, which was found to be similar across subgroups stratified by sex and BMI, and more evident among participants aged ≥44 years. No evidence of significant interactions between these three confounders and *H. pylori* infection on diabetes were found in the analyses (all *P* for interaction > 0.05).

**Table 4:** Subgroup analyses of the effects of *Helicobacter pylori* infection on diabetes according to age, sex and body mass index

Variable	Cases/participants	*H. pylori* (−)	*H. pylori* (+)	*P*	*P* for interaction
Age (years)					
≥44	2,930/30,406	1.00 (Reference)	1.25 (1.15, 1.36)	<0.001	0.914
<44	518/28,050	1.00 (Reference)	1.20(0.99, 1.47)	0.070	
Sex					
Male	2,759/36,615	1.00 (Reference)	1.26 (1.16, 1.38)	<0.001	0.593
Female	690/21,867	1.00 (Reference)	1.19 (1.01, 1.41)	0.044	
BMI (kg/m^2^)					
≥24	2,480/27,968	1.00 (Reference)	1.21 (1.11, 1.33)	<0.001	0.122
<24	778/27,838	1.00 (Reference)	1.40 (1.20, 1.64)	<0.001	

Subgroup analyses were adjusted for age, sex, body mass index (BMI), triglyceride, total cholesterol, low‐density lipoprotein, high‐density lipoprotein, uric acid, creatinine, and hypertension. *H. pylori*,* Helicobacter pylori*.

## Discussion

In the present study, it was observed that *H. pylori* infection was related to an increased risk of diabetes after adjustment for age, sex, BMI, blood lipids, uric acid, creatinine and hypertension. The present study also showed that the *H. pylori*‐positive participants were more likely to have higher levels of FPG and HbA1c after controlling for potential confounders. In addition, the present study showed that *H. pylori* infection was significantly related to diabetes in participants aged ≥44 years, but not in participants aged <44 years, although there was no evidence of effect modification. The prevalence of diabetes (5.9% vs 10.9%) and *H. pylori* infection (32.1% vs 55.8%) among Chinese adults in the present study were lower than those reported in previous investigations^3^, ^19^. As most of the participants in the present study were urban residents employed by government or enterprises, they might have relatively higher levels of education and socioeconomic status, and better sanitary conditions, which could help explain the lower prevalence of *H. pylori* infection and diabetes in our study population. In addition, the present study did not carry out the oral glucose tolerance test to diagnose diabetes, which might lead to the underestimated prevalence of diabetes.

A cross‐sectional study of 30,810 middle‐aged and older participants from China showed that individuals carrying *H. pylori* presented an increased risk of diabetes, independent of potential confounders^13^. This finding was consistent with the present results, but the study was restricted to a middle‐aged and older population. Evidence shows that *H. pylori* infection inactively occurs in young adults^20^, so it would be interesting to investigate the effect of *H. pylori* infection on diabetes among young adults. The subgroup analyses showed that *H. pylori* infection was not related to diabetes in participants aged <44 years, as Hsieh *et al*.^14^ showed in participants aged <45 years. The impact of *H. pylori* infection on diabetes in the younger population remained to be elucidated. Two cross‐sectional studies from Taiwan reported that participants carrying *H. pylori* had a higher risk of diabetes^14^, ^15^, whereas no positive association between them was observed in a Turkish study^16^. These three studies were hospital‐based, and recruited participants who underwent gastroendoscopy and gastric biopsy. We know that participants who underwent gastric biopsy were very likely patients suffering gastrointestinal diseases, potentially biasing the results. In contrast, the participants included in the present study were from the general population.

As observed in previous studies, *H. pylori* infection was closely correlated with higher levels of FPG and HbA1c, independent of potential confounders^13^, ^14^, ^21^, which was in accordance with the present findings. FPG, a frequently‐used biomarker that reflects instant plasma glucose level, could be substantially influenced by diet content and the amount of exercise before the physical examination. However, participants in the present study were asked to fast overnight and not to carry out moderate‐to‐vigorous exercise before having health checkups. Thus, diet and exercise might have little effect on the association between *H. pylori* infection and FPG in the present study. HbA1c, however, a more reliable index that reflects the long‐term average level of glycaemia^22^, ^23^, is considered more valid to evaluate the association between *H. pylori* infection and glycemic metabolism profile.

It remains to be elucidated how *H. pylori* infection contributes to diabetes, but inflammation and appetite‐related hormones are considered to be of importance. It was reported that individuals suffering *H. pylori* infection were more likely to have elevated levels of several inflammation cytokines, such as tumor necrosis factor‐α, interleukin‐6 and C‐reactive protein^24^, ^25^. These cytokines were related to insulin resistance and diabetes development^26^. In addition, Gram‐negative bacteria, such as *H. pylori*, that colonize the stomach can accelerate the secretion of lipopolysaccharide, a substance that stimulates innate inflammation processes^27^. Preceding studies documented that *H. pylori* infection affected the regulation of appetite‐related hormones, such as ghrelin and leptin^28^, ^29^. Ghrelin and leptin play a key role in energy homeostasis. Furthermore, these two hormones were reported to be closely correlated with the pathogenesis of obesity and glycometabolism^30^, ^31^, leading to the occurrence and development of diabetes consequently.

In the current study, *H. pylori* infection was significantly related to diabetes in participants aged ≥44 years, but not in participants aged <44 years, although there was no evidence showing that the interaction between *H. pylori* infection and age had an effect on diabetes development. Several studies investigated the effect of *H. pylori* infection on diabetes among middle‐aged or older individuals, and found a significant relationship between them^11^, ^13^, ^14^, which was in accordance with the present findings. Most new *H. pylori* infection occurs during childhood^32^, ^33^. Additionally, the *H. pylori* infection could perpetuate throughout a lifetime unless eradication therapy is administered^33^. Thus, younger adults usually have a shorter history of *H. pylori* infection. The long‐term cumulative effect and long history of *H. pylori* infection might help explain why *H. pylori* infection was found to be related to diabetes among participants aged ≥44 years.

In the present study, the average age of the included participants was 43.81 years. We know that most patients with type 2 diabetes mellitus are diagnosed at middle age and older. Thus, there might be a very limited number of type 1 diabetes mellitus patients in the current study. In addition, two meta‐analyses showed that *H. pylori* infection was associated with type 2 diabetes mellitus, but not type 1 diabetes mellitus^10^, ^34^. Therefore, the effect of type 1 diabetes mellitus was not considered to bias the finding of the present study that participants with a lower BMI were at higher risk of *H. pylori*‐related diabetes. As we know, overweight or obesity is an important risk factor for type 2 diabetes mellitus, and contributes greatly to its occurrence and development. Hence, overweight or obesity might mask the effect of *H. pylori* infection on diabetes in participants with BMI ≥24 kg/m^2^. Inversely, the effect of *H. pylori* could be highlighted in participants with BMI <24 kg/m^2^, leading to a higher risk of *H. pylori*‐related diabetes in participants with a lower BMI.

We hypothesized that *H. pylori* infection was early‐onset in the present study, as people generally become infected with *H. pylori* during childhood^32^, ^33^. However, if the diabetes was early‐onset, we could not exclude the possibility that diabetes increased the risk of *H. pylori* infection. One explanation was that diabetes could impair the body's cellular and humoral immunity^35^, resulting in increased susceptibility to bacterial infection. Another explanation suggested that diabetes could reduce gastric motility and acid secretion^36^, ^37^, consequently leading to vulnerability to *H. pylori* infection. Therefore, it would be interesting to validate the bidirectional association between *H. pylori* infection and diabetes in the future.

The present study had several strengthens. First of all, our study investigated the effect of *H. pylori* infection on diabetes among young adults in mainland China. Second, we included a large sample from the general population and adjusted for important risk factors, which make the results more reliable and convincing. Third, we used the ^13^C‐urea breath test to determine *H. pylori* status. This is a non‐invasive and effective diagnostic method for *H. pylori*, with high sensitivity and specificity^38^, ^39^. As we know, this is the first study to examine the association of diabetes with *H. pylori* infection using the ^13^C‐urea breath test. Fourth, FPG and HbA1c were used to describe the glycemic metabolism profiles, indicating that we evaluated the association of *H. pylori* infection with both instant and long‐term average levels of glycemia.

There were also some limitations. First, the causal relationship of *H. pylori* infection and diabetes could not be determined due to the cross‐sectional design. However, people generally become infected with *H. pylori* during childhood^32^, ^33^. Therefore, participants with both type 2 diabetes mellitus and *H. pylori* infection might suffer *H. pylori* infection at an earlier age. Second, although we adjusted for several major risk factors, other potential confounders that we did not measure, such as the use of antibiotics, family history of diabetes, waist circumference, alcohol consumption and smoking, might bias the results. Third, the participants were recruited from a physical examination center and might not represent all the Chinese adults, which limited the extrapolation of the present findings. Fourth, we did not carry out the validation study of the ^13^C‐urea breath test by gold standard diagnostic tools, such as gastroscopy and subsequent biopsy, bacterial culture, and anti‐*H. pylori*. However, previous studies have shown excellent agreement between the ^13^C‐urea breath test and gastroscopy with biopsy^40^, ^41^, indicating that the ^13^C‐urea breath test has high diagnostic accuracy and good performance.

In summary, the present findings suggest that *H. pylori* infection is independently related to a higher risk of diabetes among Chinese adults. More attention should be paid to adults with *H. pylori* infection for effective prevention of their diabetes. The prevention and treatment of *H. pylori* infection might not only decrease the burden of gastrointestinal diseases, but also delay the development of diabetes, therefore reducing the heavy burden of diabetes. Well‐designed prospective studies are warranted to confirm the effect of *H. pylori* infection on the incident risk of diabetes and corresponding confounders.

## Disclosure

The authors declare no conflict of interest.
